# Vertical leaping mechanics of the Lesser Egyptian Jerboa reveal specialization for maneuverability rather than elastic energy storage

**DOI:** 10.1186/s12983-017-0215-z

**Published:** 2017-07-03

**Authors:** Talia Y. Moore, Alberto M. Rivera, Andrew A. Biewener

**Affiliations:** 1 Concord Field Station, Harvard University, 100 Old Causeway Road, Bedford, MA 01730 USA; 2 University of Michigan, Museum of Zoology and Department of Ecology and Evolutionary Biology, Ruthven Museum, 1109 Geddes Ave, Ann Arbor, MI 48109 USA

**Keywords:** Jerboa, Inverse dynamics, Muscle-tendon stresses, Ricochetal bipedal locomotion

## Abstract

**Background:**

Numerous historical descriptions of the Lesser Egyptian jerboa, *Jaculus jaculus*, a small bipedal mammal with elongate hindlimbs, make special note of their extraordinary leaping ability. We observed jerboa locomotion in a laboratory setting and performed inverse dynamics analysis to understand how this small rodent generates such impressive leaps. We combined kinematic data from video, kinetic data from a force platform, and morphometric data from dissections to calculate the relative contributions of each hindlimb muscle and tendon to the total movement.

**Results:**

Jerboas leapt in excess of 10 times their hip height. At the maximum recorded leap height (not the maximum observed leap height), peak moments for metatarso-phalangeal, ankle, knee, and hip joints were 13.1, 58.4, 65.1, and 66.9 Nmm, respectively. Muscles acting at the ankle joint contributed the most work (mean 231.6 mJ / kg Body Mass) to produce the energy of vertical leaping, while muscles acting at the metatarso-phalangeal joint produced the most stress (peak 317.1 kPa). The plantaris, digital flexors, and gastrocnemius tendons encountered peak stresses of 25.6, 19.1, and 6.0 MPa, respectively, transmitting the forces of their corresponding muscles (peak force 3.3, 2.0, and 3.8 N, respectively). Notably, we found that the mean elastic energy recovered in the primary tendons of both hindlimbs comprised on average only 4.4% of the energy of the associated leap.

**Conclusions:**

The limited use of tendon elastic energy storage in the jerboa parallels the morphologically similar heteromyid kangaroo rat, *Dipodomys spectabilis*. When compared to larger saltatory kangaroos and wallabies that sustain hopping over longer periods of time, these small saltatory rodents store and recover less elastic strain energy in their tendons. The large contribution of muscle work, rather than elastic strain energy, to the vertical leap suggests that the fitness benefit of rapid acceleration for predator avoidance dominated over the need to enhance locomotor economy in the evolutionary history of jerboas.

**Electronic supplementary material:**

The online version of this article (doi:10.1186/s12983-017-0215-z) contains supplementary material, which is available to authorized users.

## Background

Jerboas are small bipedal rodents native to the deserts of northern Africa and Eurasia that use erratic hopping locomotion, often called ricochetal saltation, to navigate their arid habitat, forage for scarce resources, and escape from predators. They constantly switch between hopping, running, turning, and leaping vertically as they move on the shifting sand [1, 2]. The inherently variable locomotion of jerboas presents a challenge for biomechanical analyses commonly designed for steady-state locomotion [2, 3]. Fortunately, jerboas perform a pronounced vertical leap to escape predation that can be elicited in a laboratory setting [4]. These escape leaps enable jerboas to forage in open areas where the risk of avian predation is higher [2, 5]. Vertical leaping is therefore a broadly useful behavior to examine in jerboas, since leaps to escape predators likely approach maximal performance, and leaping is relevant to jerboa survival.

Understanding how animals use their musculoskeletal system to generate a broad range of locomotor behaviors informs our understanding of how evolution has shaped locomotor performance. Since muscles require metabolic energy to actively contract, whereas tendons are passively elastic, determining the relative mechanical energy contributions of muscles and tendons to locomotor movements can help to inform predictions of locomotor endurance. Cursorial animals adapted for sustained and repetitive locomotion tend to have greater tendon elastic energy storage [6]; energy recovered from tendons offsets the amount of muscle work required over the course of a stride, significantly lowering cost of transport. For example, elastic energy recovery provides 40–70% of the total center of mass (CoM) mechanical energy during sustained hopping in bipedal red kangaroos (*Macropus rufus*) [7] and 36% of CoM mechanical energy during galloping in horses (*Equus ferus caballus*) [8]. Both of these animals are able to sustain high speed locomotion over long time periods because the passive energy storage in tendons decreases the need for muscle work to move the animal’s body during each step.

Although tendon energy storage and recovery can provide more economical locomotion, the lengthening of compliant tendons likely slows the ability of muscles to produce limb movement. Therefore, small prey animals requiring quick accelerations to escape predator threats tend to use less tendon energy storage in their locomotion, allowing muscle-tendon units in their hindlimbs to shorten more quickly. For example, the kangaroo rat, *Dipodomys spectabilis*, which reflexively leaps in response to the vibrations emitted by their predators [9], elastically recovers only 14% of the mechanical energy in tendons during forward hopping [10] and 21% during leaping [11]. Despite considerable phylogenetic distance between kangaroo rats and jerboas [12], the morphological and behavioral similarity between the species lead us to hypothesize that jerboas, as exemplified by *J. jaculus*, similar to kangaroo rats, store only a small amount of elastic energy in their tendons during vertical leaping.

For non-steady-state locomotion, elastic energy can be gradually stored in tendons as muscles contract and returned rapidly to amplify a muscle-tendon unit’s capacity to produce power [13]. Because this mechanism requires preparation time to preload the tendons, power amplification is most often associated with isolated jumps from a stationary position. Several invertebrates use power amplification and specialized ratcheting morphology to achieve incredible leaps, up to 100x body length (summarized in [14]). Power amplification has also been demonstrated to enable frog leaps of up to 8x their body length [15]. However, it is unknown whether jerboas are able to use power amplification to enhance their vertical leaping performance.

In this study, following similar methods used to study red kangaroos [7], we used joint moment analysis based on measurements of 2D limb kinematics and ground reaction forces (GRFs) to calculate the relative contributions of jerboa hindlimb muscles and tendons to produce the energy of vertical leaping. In this study we build upon previous descriptions of jerboa hindlimb morphology [4, 16], with detailed dissections of hindlimb muscle and tendon architecture to determine the role of each hindlimb muscle-tendon element in the execution of vertical leaping.

## Methods

### Animals

We tested five *J. jaculus* (four males, one female) from the colony at the Concord Field Station that were originally captured from the wild in Egypt. Their masses ranged from 53 to 74 g. More animals were tested, but were non-responsive to the stimulus and refused to leap, possibly due to lack of motivation. To estimate the morphological measurements of each subject, we dissected three other jerboas that were euthanized for other studies and assumed geometric scaling between individuals. Before experimentation, we shaved the jerboas’ legs and used a non-toxic marker to indicate joint positions. All animal care and use protocols were approved by the Harvard Faculty of Arts and Sciences Institutional Animal Care and Use Committee (IACUC) and the United States Department of Agriculture.

### Experimental setup

At the start of each trial we placed the animal in a wood and plexiglas structure (103 × 15 × 15 cm) on a force platform. Data were initially collected from a 2-axis (vertical and fore-aft) custom-made (6 × 12 cm) strain gauge force platform [17], which fed into a data acquisition system (BioPac MP150). Due to damage of this force platform, subsequent recordings comprising an additional dataset were collected with a rigid plate mounted on a load cell with 6 degrees of freedom (ATI Nano43). A meter stick attached to the back of the enclosure indicated the maximum height of each leap. We used quick bursts of compressed air to motivate the animals to leap. An additional file shows a representative trial (Additional file 1: Figure S1).

To film each trial, we lit the area with a 500 W light (Omni Lowell) and placed two high speed cameras in front of the enclosure to film the leaps in lateral view. In the original dataset, one camera (Casio ZR100) with a wide angle lens was positioned to film the entirety of each leap at 240 fps and provided maximum leap height of each trial. The other camera (IDT NR5) equipped with a zoom lens was positioned to provide a smaller field of view that allowed detailed motion of joint positions to be determined at 250 fps during limb contact and takeoff from the force platform. In the additional trials, one camera (GoPro Hero 3+) recorded at 120 fps and provided a view of how the feet are placed on the force platform. The other camera (Photron SA3) equipped with the zoom lens recorded at 250 fps to capture both detailed motion of joint positions and maximum jump height. For this analysis, we selected only leaps in which one or both feet were in contact with only the force platform, with the animal’s mediolateral body axis oriented parallel to the camera filming axis. We assumed that the animal leapt with equal force on both legs, and divided total ground reaction force in half to compute single limb forces for trials with both feet on the force platform. Positions of the joints (metatarso-phalangeal, ankle, knee, and hip), eye, and base of tail were tracked using custom tracking software (DLTdv5 Matlab program) [18].

### Inverse dynamics

We used an inverse dynamics approach that ignored inertial and gravitational segmental moment effects to calculate the total agonist muscle force required at each joint (from distal to proximal, using a linked-segment model) to resist the moment produced by the ground reaction force (GRF) in each frame of video. The GRF moment is the cross-product of the GRF originating from the center of pressure (CoP) at the base of the foot measured by the force platform with respect to the joint’s center of rotation, which defines the GRF moment arm [17]. Because ground reaction forces had negligible mediolateral and fore-aft horizontal components, we estimated each GRF moment arm to be a horizontal distance between the joint and CoP.

Due to vibrations arising from resonance of the fore and aft vertical force sensors, we were unable to obtain reliable CoP measurements for the initial force platform. High-speed video showed that the foot lifted off and lost contact with the ground incrementally from the MTP (metatarso-phalangeal) joint to the toes, indicating that anterior movement of the CoP is greatest near the end of takeoff. We therefore estimated the position of the CoP as initially being 25% of the distance from the MTP to the toes and moving exponentially in the x-direction towards the distal end of digit III over the course of leap takeoff.

The data presented here are based on a model in which CoP distance from the MTP, *c*, is defined as *c*_*t*_=*r*/4+(3*r*/4)∗*e* ^*dt*−*d*^, where *r* is the distance between the MTP and the toe, *d* is the duration of the takeoff in frames of high-speed video, and *d*_*t*_ is the given frame for which *c*_*t*_ is calculated (Additional file 2: Figure S2). Although changing the CoP movement model has some effect on joint torques (Additional file 3: Table S1), especially at the MTP and hip, the general pattern of joint torques remains robust (Additional file 4: Figure S3).

GRF moments at each joint are resisted by the contraction of muscles that cross the joint, creating a counteracting muscle joint moment. At each joint, we assumed that each agonist muscle exerts a force proportional to its physiological cross-sectional area (PCSA), or similar peak stress. We calculated PCSA using the equation

1 $$ PCSA = \frac{mass * cos(\phi)}{\rho_{m} * fiber length}   $$

where *ϕ* is pennation angle, and *ρ*_*m*_ is the density of muscle (1060*k* *g*/*m* ^3^ according to [19]). Additionally, we assumed no co-contraction of antagonistic muscle pairs, except in the cases of biarticulate muscles spanning two joints.

**Fig. 1:**
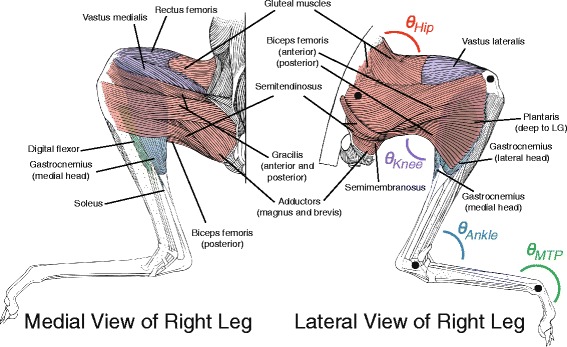
Diagram of the muscles considered in this analysis. Muscles are colored according to the joint at which they primarily act. *Joint angles* are defined such that *θ* increases as the arc represented elongates, and *black circles* indicate approximate centers of rotation for each joint. Anatomical sketches of limb muscles are adapted from drawings by Howell [16]

The muscles counteracting the GRF moment at the most distal joint, the MTP, are the digital flexors and plantaris (Fig. 1, in green); whereas, plantarflexor muscles — the plantaris, soleus, and gastrocnemius (Fig. 1, in blue) — resist the ankle GRF joint moment (in jerboas, the moment arm of the digital flexors is close to zero at the ankle). Because the plantaris muscle exerts a moment at both the MTP and ankle joints, plantaris muscle-tendon force was first calculated at the MTP joint, then subtracted from the total ankle plantarflexor muscle moment (*M*_*A*_−*F*_*plant*_×*r*_*plant*_), leaving the remainder of the moment to be generated by the gastrocnemius and the soleus. At the knee joint, rectus femoris, vastus lateralis, vastus medialis, and vastus intermedius (i.e. quadriceps) all resist the GRF knee flexor moment (Fig. 1, in purple), in addition to flexor moments produced by the bi-articular gastrocnemius and tri-articular plantaris that have origins from the femoral epicondyles. Thus, the quadriceps knee extensors balance the sum of the GRF moment at the knee and the opposing flexor moments from the gastrocnemius and plantaris: (*M*_*K*_+*F*_*gast*_×*r*_*gast*_+*F*_*plant*_×*r*_*plant*_). Similarly, the rectus femoris applies an opposing flexor moment at the hip. The hip extensors considered to resist hip flexor moments were the biceps femoris, gluteus muscles (medius, medialis, and minimus), adductor magnus, and semitendinosus (Fig. 1, in red). These muscles resist the GRF flexor moment at the hip, in addition to that produced by rectus femoris at the knee (*M*_*H*_+*F*_*recF*_×*r*_*recF*_).

Joint angles (Fig. 3 a) as defined in Fig. 1 (labeled *θ*) were obtained using the following equation:

2 $$ \theta_{1,2} = abs({a}cos(limb \;{element}_{1} \cdot limb \;{element}_{2}))   $$

**Fig. 2:**
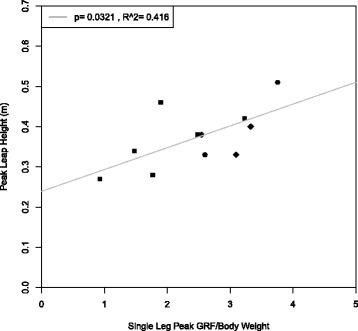
Height versus normalized force for each trial. The *x-axis* represents the maximum force recorded during each leap divided by body weight. The *y-axis* is a conservative estimate of peak CoM height. Of the data collected, only 11 leaps from three individuals involved no contact with the side walls and are included in this figure

**Fig. 3:**
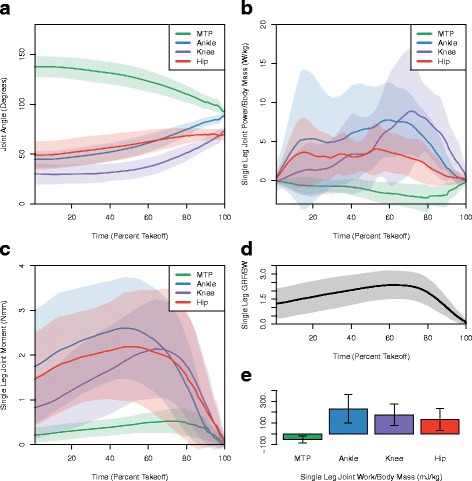
**a** Mean joint angle, **b** mass-specific power, **c** moment, **d** ground reaction force divided by body-weight, and **e** work for a single leg throughout a trial. Trials were scaled to be the same percentage of takeoff. The *shaded area* corresponds to the standard deviation on either side of the mean in bold

where · indicates the dot product. Joint angles were differentiated to obtain angular velocity and multiplied by the joint moment to calculate joint power. Joint power was integrated over time to calculate net joint work over the takeoff phase of the leap. Hip angle was not available during the entirety of all trials, due to the anterior portion of the animal occasionally leaving the field of view near the end of the trial. Trials with complete hip angle data showed that hip angle remained relatively constant throughout the trial. Therefore, to obtain hip joint work values, the hip joint angle was assumed to remain constant (i.e. no additional joint work) throughout the remainder of the trial once it disappeared from the camera’s field of view.

From the muscle-tendon force data, we calculated the strain energy storage in the digital flexor, plantaris, and Achilles tendons. Tendon stress was calculated by dividing the force by tendon cross-sectional area. Tendon cross-sectional area was calculated using the following equation:

3 $$ CSA = \frac{mass}{\rho_{t} * length}   $$

where the density of tendon (*ρ*_*t*_) is 1120*k* *g*/*m* ^3^ [20]. Strain is stress divided by the tendon elastic modulus. We used a value of 1.0 GPa [21–23], which approximates the average modulus over a tendon strain range of 0–5% [8]. Overall tendon length change was calculated as strain multiplied by resting tendon length (measured during dissection from muscle-tendon unit as origin to insertion minus muscle fascicle length). Tendon elastic energy was then calculated assuming Hookean behavior as: $ W = \frac {1}{2} F\Delta L$. Although this assumption ignores the “toe” region of the J-shaped tendon elasticity curve, our use of a lower elastic modulus (1.0 GPa) compared with the modulus for the linear stress-strain region (≈1.2 GPa) helps to correct for overestimates based on an assumption of linear elasticity [8]. Because tendon resilience is ≈ 93% [24, 25], we multiplied tendon energy storage by 0.93 to estimate the energy recovered that could help to power the animal’s leap. We compared the tendon energy in both limbs to the total energy of the leap, as determined by potential energy at maximum leap height, to compute tendon energy recovery for each trial. To provide a conservative estimation of the CoM location, we recorded the location of the rump behind the hip at maximum leap height to calculate potential energy. Unless noted otherwise, data are reported as mean ± SD.

## Results

We analyzed 36 trials from five jerboas (2–14 leaps per animal). Eleven trials from three individuals involved no contact with the sides of the enclosure, and could therefore be used to determine maximum leap height and total energy of the leap. In each figure, data points for each individual have the same shape.

### Leap patterns

Jerboas leapt to a mean recorded height of 0.37 m, with a maximum leap height exceeding 0.60 m (Leap height vs peak GRF was not included for the highest trials, as jerboas truncated their leaps by gripping onto the wall and escaping from the enclosure. Experimenters chose to recapture the animal in lieu of being able to save the recorded data for those trials). The highest leaps were approximately 10 times hip height at mid-stance during forward locomotion (6.1 cm, calculated from forward locomotion data collected for [2]). Average peak single-leg GRF was 2.6 (N/body weights) with a maximum of 4.5 (N/body weights). A positive correlation between maximum leap height and peak vertical GRF was observed (*p* = 0.03, *R* ^2^= 0.42, Fig. 2). Few leaps were immediate takeoffs from a previous landing. Oftentimes jerboas would perform multiple leaps in succession. However, due to there being a few seconds between each leap (see Additional file 1: Figure S1), countermovement leaps were rarely observed. The highest leaps, both in our dataset and those not saved and analyzed, were often the first or the only leap in a series.

### Muscle-tendon architecture

Muscle and tendon measurements are presented in Tables 1 and 2. MTP (plantar) flexors accounted for 5.4% of the total hindlimb “extensor” muscle mass (for multiarticular muscles, muscle mass distribution was categorized based on the more distal joint across which the muscle acts), with ankle extensors being 14.1%, knee extensors 24.1%, and hip extensors 56.5% of total extensor muscle mass. As expected for fast-moving limbs, muscle mass decreased in the more distal limb segments, decreasing the moment of inertia of the limb with respect to the hip. In contrast, the cross-sectional area of the MTP flexors, ankle, knee, and hip extensors accounted for 9.0, 33.9, 25.8, and 31.2% of the total hindlimb muscle cross-sectional area, respectively. Because force generation is proportional to muscle cross-sectional area, ankle and MTP plantarflexors would be expected to contribute more force with respect to their mass.

**Table 1:** Hindlimb muscle morphometric data geometrically scaled for a jerboa of mass 62.72g

Muscle	Mass	Fiber length	Pennation angle	MTP arm	Ankle arm	Knee arm	Hip arm
Plantaris	0.10	7.10	8	1.15	4.52	1.96	-
Digital Flexors	0.06	7.25	15	1.08	1.58	-	-
Medial Gastroc	0.20	4.39	15	-	4.52	2.02	-
Lateral Gastroc	0.22	5.15	18	-	4.52	2.02	-
Soleus	0.02	7.16	5	-	4.47	-	-
Rectus Femoris	0.18	9.35	16	-	-	3.33	5.75
Vastus Lateralis	0.43	13.21	11	-	-	3.33	-
Vastus Medialis	0.13	9.98	10	-	-	3.33	-
Vastus Intermedius	0.02	5.30	5	-	-	3.33	-
Biceps Femoris	0.87	32.26	10	-	-	-	5.79
Gluteus Medius	0.19	11.23	0	-	-	-	3.42
Gluteus Medialis	0.02	6.65	0	-	-	-	3.42
Gluteus Minimus	0.06	8.63	0	-	-	-	3.42
Adductor Magnus	0.56	23.01	3	-	-	-	10.52
Semitendinosus	0.07	23.46	3	-	-	-	11.32

Muscle masses are shown in grams, fiber length and moment arms are shown in millimeters, and muscle pennation angles are shown in degrees

**Table 2:** Morphometric data for hindlimb tendons geometrically scaled for a jerboa of mass 62.72 g

Tendon	Mass	Length	CSA	Flexor moment arm	Extensor moment arm
Plantaris	0.006	46.688	0.119	1.900	-
Digital Flexor	0.014	47.050	0.271	1.900	-
Achilles	0.017	19.906	0.784	-	4.820

Tendon masses are in grams, lengths and moment arms are in millimeters, cross-sectional areas are in square millimeters

### Joint work, muscle stress, and force

**Fig. 4:**
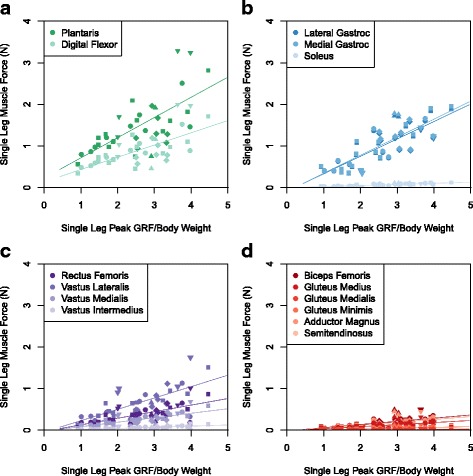
Single leg muscle forces are plotted against the corresponding peak ground reaction forces for each trial. Data from a single individual is represented by a unique symbol. Data from all individuals were grouped together to calculate the trendline for each muscle. **a** Metatarso-phalangeal flexors. **b** Ankle extensors (synergistic plantaris forces not pictured). **c** Knee extensors (antagonistic gastrocnemius force not pictured). **d** Hip extensors; antagonistic rectus femoris not pictured

MTP joint angle decreased (dorsi-flexed) throughout takeoff from the ground (Fig. 3 a), indicating negative MTP joint work during jump takeoff (-52.4 ± 31.6 mJ /kg body mass, Fig. 3 e). MTP plantar-flexor muscles exerted mean peak stresses of 132.8 kPa; much higher than the stresses exerted by muscles at other joints. The maximum muscle stress recorded (317.1 kPa in the plantaris) was less than the peak muscle stress recorded in kangaroo rat ankle extensors during vertical leaping (350 kPa) [11]. However, greater muscle stresses were likely achieved in the higher leaps not analyzed (due to animals escaping the leaping enclosure and lost data). Peak force generated by the plantaris was 3.28 N, which was the highest force produced by any single muscle belly (Fig. 4 a and Table 3). For a 64 g jerboa weighing 0.6 N, maximum force generated by the plantaris therefore exceeded five times the animal’s weight.

**Table 3:** Least-squares regression statistics for trendlines in Fig. 4

Muscle	*p* value	*F* statistic	Adjusted *R* ^2^
Plantaris	0.012	7.48	0.213
Digital Flexor	0.012	7.48	0.213
Lateral Gastrocnemius	3.350e-8	65.86	0.730
Medial Gastrocnemius	1.347e-8	73.09	0.750
Soleus	1.347e-8	73.09	0.750
Rectus Femoris	0.001	14.86	0.366
Vastus Lateralis	0.001	14.86	0.366
Vastus Medialis	0.001	14.86	0.366
Vastus Intermedius	0.001	14.86	0.366
Biceps Femoris	1.052e-5	31.42	0.559
Gluteus Medius	1.052e-5	31.42	0.559
Gluteus Medialis	1.052e-5	31.42	0.559
Gluteus Minimus	1.052e-5	31.42	0.559
Adductor Magnus	1.052e-5	31.42	0.559
Semitendinosus	1.052e-5	31.42	0.559

Work produced at the ankle joint exceeded work at any other joint, with an average of 231.6 ± 132.0 mJ/kg body mass (Fig. 4 b). Ankle plantarflexors exerted mean peak stresses of 30.8 kPa, with a maximum stress of 62.2 kPa. Similar PCSA values for lateral and medial heads of the gastrocnemius resulted in our estimate of nearly identical forces at these two muscles (Fig. 4 b). The maximum force produced by the lateral gastrocnemius head was 1.9 N, with the maximum force produced by both heads being 3.8 N. Due to its much smaller PCSA, the soleus contributed very little to the ankle moment, exerting an estimated maximum force of 0.1 N.

An average of 175.4 ± 99.6 mJ/kg body mass of work was produced at the knee (Fig. 4 c); considerably less than expected based on the cross-sectional area of the knee flexors relative to the ankle extensors and MTP plantarflexors (Table 1). As a group, the quadriceps produced average peak stresses of 17.0 kPa, with a maximum of 42.7 kPa. Given its larger size, the vastus lateralis generated the greatest estimated peak force at the knee (mean 0.7 N, max 1.8 N, Fig. 4 c). As the smallest of the quadriceps, the vastus intermedius contributed the least force to the knee extensor moment (mean 0.1 N, max 0.2 N, Fig. 4 c).

An average of 132.9 ± 103.4 mJ/kg body mass of work was produced at the hip, contributing the least amount of positive work relative to the knee and ankle joints to leap potential energy (Fig. 4 d). Hip adductors produced an average peak stress of 6.3 kPa and a maximum stress of 14.1 kPa. The greatest peak forces at the hip were produced by the biceps femoris (mean 0.2 N, max 0.5 N, Fig. 4 d). Although the hip extensors have a greater total cross-sectional area (Table 1) and a greater number of muscles in comparison to agonist extensor and plantarflexor groups at more distal joints, the hip extensors contributed less net positive work toward the vertical leap than the ankle or knee joints due to the hip angle remaining relatively constant throughout the takeoff of each trial (Fig. 4 d).

Power produced by muscles acting at each joint peaked at different times during leap takeoff (Fig. 3 b). The MTP moment was small throughout the takeoff, due to the close proximity of the MTP joint to the CoP. Consequently, the muscles acting at the MTP produced small amounts of negative power (due to MTP dorsiflexion) throughout the takeoff (Fig. 3 b). The ankle, knee, and hip moments gradually increased until 60–70% of takeoff, and then decreased rapidly after peak GRF, toward the end of takeoff, as the animal left the ground and rose into the air (Fig. 3 c). Joint power generated by the ankle and hip exhibited two peaks, one at 15% takeoff, and one 60% takeoff (Fig. 3 b). On the other hand, the joint power generated by muscles acting at at the knee had a single peak, with the knee occurring at 80% takeoff (Fig. 3 b).

### Tendon energy recovery

**Fig. 5:**
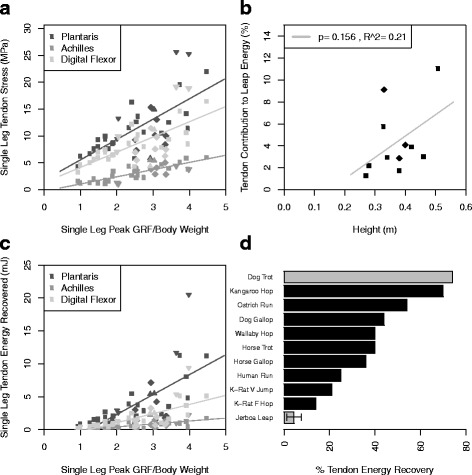
Tendon stress and elastic energy storage. Data from a single individual is represented by a unique symbol. Data from all individuals were grouped together to calculate the trendlines. **a** Tendon stress as a function of ground reaction force. Plantaris tendon *p*=0.001, *F*=16.13, adjusted *R* ^2^=0.387; digital flexor tendon *p*=0.001, *F*=16.13, adjusted *R* ^2^=0.387; achilles tendon *p*=1.299*e*−7, *F*=56.12, adjusted *R* ^2^=0.697. **b** Tendon energy contribution (for two hindlimbs) to the total energy of the leap, calculated from the potential energy at peak leap height. **c** Single leg tendon energy contributions as a function of ground reaction force. Plantaris tendon *p*=0.0002, *F*=18.52, adjusted *R* ^2^=0.422; digital flexor tendon *p*=0.0002, *F*=18.52, adjusted *R* ^2^=0.422; achilles tendon *p*=1.341*e*−6, *F*=41.82, adjusted *R* ^2^=0.630. **d** Jerboa tendon energy contribution to total limb mechanical work (forward locomotion in all cited studies, except for kangaroo rat vertical jumping) or CoM work (vertical leaping in jerboas) compared to other species. Dog data from [41], kangaroo and wallaby data from [7], ostrich and human data from [42], horse data from [8], kangaroo rat forward hopping data from [10]. Tendon energy recovery in the kangaroo rat during vertical jumping was estimated to be 8.6-fold greater relative to hopping, calculated by comparing muscle-tendon stresses during forward hopping versus vertical jumping [10, 11]

We analyzed the plantaris, digital flexor, and Achilles tendons for their contribution to strain energy storage and recovery during leaping, as these are the largest tendons in the hindlimbs and attach to muscles producing the greatest force. The plantaris tendon experienced the greatest peak stresses (mean 11.6, max 25.6 MPa), and the Achilles experienced the least (mean 3.2, max 6.0 MPa), despite transmitting greater total force from both heads of the gastrocnemius (Figs. 5 a, 4 b). All tendon stresses were well within the tensile strength of vertebrate tendon, ≈100 MPa [21], and had a minimum safety factor of 3.9.

The low tendon stresses resulted in very small amounts of energy being recovered from the tendons. The maximum energy contribution of a single tendon throughout a leap was approximately 20 mJ, and the maximum energy recovery throughout a leap from all tendons in both hindlimbs was 64.2 mJ (Fig. 5 c), in a trial without maximum jump height. The maximum recorded leap energy was estimated to be 314.9 mJ, with the tendons contributing 22.0 mJ (14.3% energy recovery) for that trial. Tendon contributions to total leap energy for both hindlimbs averaged 4.4% ± 3.1% (Fig. 5 d) and showed no significant relationship with peak leap height, although the lack of significance may be due to small sample size (Fig. 5 b).

## Discussion

Muscle forces in this paper have been analyzed under a number of assumptions, both to simplify the analysis and to enable direct comparison to previous studies of jumping mammals. Electromyographic recordings in future studies could determine whether co-contraction of antagonistic muscles would need to be incorporated into the model, which would increase the estimated force produced by the muscles. Similarly, accounting for force-length (F-L) and force-velocity (F-V) effects in future analyses of jerboa leaping would be useful, if such analyses were related to the F-L and F-V measurements of key hindlimb muscles. Based on our study, the gastrocnemius and plantaris muscles would be most important to assess, as our inverse dynamics analysis indicates that these muscles generate the greatest work during leaping. Finally, it would be of interest to know the fiber type distributions for these muscles, but such data are not currently available, other than for the soleus [26, 27], which is comprised of type I fibers. However, our analysis shows that the soleus is extremely small and cannot contribute much work to leaping. Thus, further experimentation and muscle modeling would enable a more detailed analysis, though we believe that these additional considerations would minimally affect the significance of the results presented here.

Studying jerboa vertical leaping under controlled laboratory conditions represents an important first step in understanding how and why these small mammals generate some of the highest leaps (relative to hip height) of most mammals [28, 29]. Although we observed leaps in excess of 10 times hip height in the laboratory, observations of jerboas in the wild suggest that jerboas are capable of more extreme leaping maneuvers [30]. Indeed, the low values of mean muscle and tendon stresses we calculated here suggest a greater capacity for leaping and accelerative maneuvering than we observed in the laboratory. The restrictive artificial enclosure, including the solid substrate, likely limited the jerboas’ motivation and performance. That field performance may substantially exceed laboratory performance has been recorded in other species, and highlights the importance of identifying and quantifying those stimuli that motivate animal locomotion [31, 32]. Despite the somewhat subdued behaviors exhibited by jerboas in laboratory settings, the mechanistic understanding gained from a biomechanical analysis of leaping performance helps to predict the limits of their performance for other behaviors and the selective pressures favoring the evolution of their locomotion.

During leaping, we observed a consistent pattern of peak hip extension and work early in takeoff, with little change throughout the rest of takeoff. This likely elevates the CoM to minimize pitch instability of the trunk during subsequent knee and ankle power output. The early peak of jerboa hip power matches other leaping vertebrates, such as frogs, galagos, humans, and cats [28, 33–35]. Lizards leaping from substrates with variable friction provide further evidence that trunk pitch is important to a successful leap — perturbations to trunk pitch during takeoff are rapidly corrected with inertial movements of the tail [36]. Finally, in contrast to power generated at the hip, knee, and ankle joints, negative power (energy absorption) occurs at the MTP joint during leaping. Interestingly, this pattern parallels MTP energy absorption in wallabies during acceleration [37] and in goats during incline locomotion [38], and may reflect the biarticular transfer of energy from the MTP joint via the plantaris tendon to contribute power for ankle extension.

The contribution of jerboa tendon elastic energy recovery to CoM work during leaping is surprisingly low, even when compared to tendon elastic energy recovery in kangaroo rats during forward hopping and vertical leaping [10, 11], despite these animals being morphologically and behaviorally convergent. Unlike small bipedal rodents, kangaroos have thinner tendons (relative to body size) that store and return substantially more elastic energy, enabling them to perform sustained bouts of steady-state cursorial locomotion; while simultaneously hindering accelerative ability, which is likely unnecessary due to their lack of consistent predation pressure [39, 40]. For both bipedal and quadrupedal cursorial animals, even small stride-to-stride energy savings can add up to substantial energy savings over time, reducing the cost of foraging. Dogs, horses, kangaroos, and ostriches can recycle 36–74% of their total limb mechanical work by storing energy elastically in tendons [7, 8, 41, 42]. In comparison, jerboas and kangaroo rats recover far less energy compared with the CoM work performed during locomotion and leaping (Fig. 5 d), and rely on acceleration capacity to escape predation [43]. Thus, muscle-tendon morphology suggests a significant difference in the ecological context and selective pressures encountered by small and large bipedal hopping mammals.

While energetically costly, locomotion that is predominantly powered by muscular contraction has the benefit of producing rapid changes in movement, or a high acceleration capacity. Because compliant tendons result in greater stretch for a given amount of force, it requires a muscle to shorten a greater distance and (for a given shortening rate) a longer time to produce movement at a joint. Therefore, reduced tendon stretch and energy storage can be advantageous, especially for prey animals that must produce rapid joint movements to change speed or direction for predator evasion [44]. Because of the high energetic cost, this strategy would be most appropriate for evading predators that are committed to a single strike, rather than pursuit over long distances.

It is difficult to discern whether the small size of jerboas and kangaroo rats constrains their tendon morphology, and thus their capacity for elastic energy storage. Biewener and Bertram [39] argue that because tendons are generally thicker than expected based on strength [45], kangaroo tendons have evolved to be thinner than expected for their body size to favor elastic energy storage at the expense of a reduced acceleration ability and control of rapid movements. However, it is unclear if small jerboa-sized mammals also have the ability to evolve thinner tendons for enhanced elastic energy recovery. Kangaroo rat tendons are thicker than expected given geometric similarity, and would require ≈80% reduction in cross-sectional area to confer elastic energy recovery equivalent to a kangaroo or wallaby [10]. Relatively few biomechanical analyses have examined the terrestrial locomotion of quadrupedal mammals smaller than 1 kg, because most small mammals (including the quadrupedal ancestors of jerboas) are ambulatory generalists with fewer less obvious biomechanical specializations [46, 47]. Elephant shrews (*Elephantulus spp.*, Macroscelidae) would provide the most informative comparison, as they are the only identified group of micro-cursorial quadrupedal mammals [48]. Evidence of thinner tendons than expected by geometric similarity in elephant shrew hindlimbs would suggest that animals of small size may not be constrained to have stiff tendons with low elastic energy storage. This would lend support to the argument that, jerboas and kangaroo rats likely encountered selection favoring greater tendon thickness and force transmission, allowing for rapid accelerative movements.

The low level of tendon strain computed in this analysis suggests that jerboas do not rely on power amplification to achieve the leaps that we recorded. Power amplification has been indirectly demonstrated to occur in other mammals during jumping, such as rock wallabies and galagos, which frequently move over irregular and discontinuous locomotor substrates [28, 49]. Jumping that is predominantly powered by muscle contraction has the advantage of requiring no extra time to preload the tendon, thus making it possible to produce a more rapid leaping movement. Thus, muscle-powered leaps have the potential to enhance the three-dimensional complexity of a trajectory, which is important for evading single-strike predators on a continuous locomotor matrix [25, 44]. Since jerboas and kangaroo rats are only found in continuous desert environments, leaping that is predominantly powered by muscle contraction likely provides a greater advantage to their predator evasion ability than leaping via power amplification from their tendons.

## Conclusion

Our results show that the hindlimb morphology of jerboas, much like kangaroo rats, favors the rapid generation of large ground reaction forces during leaping by reliance on muscle work rather than elastic energy recovery to power acceleration and movement. Such short bouts of rapid leaping would be particularly well suited to evading single-strike predators, especially in desert ecosystems where sympatric quadrupedal rodents are at greater risk for predation due to moving with lower velocities and less unpredictable trajectories [2]. Future studies of biomechanical performance in a field setting will provide important insight into the evolutionary and ecological context of this spectacular leaping rodent.

## Additional files

Additional file 1 **Figure S1.** A video of a sample vertical leap. The jerboa is standing upon a 2-axis force plate, inside of a vertical trap. In the presence of increased air flow, the jerboa leaps vertically. (MOV 641 kb)

Additional file 2 **Figure S2.** Center of Pressure Sensitivity Analysis. The x-distance between the CoP and the toe through time, in a representative trial. The x-distance between the MTP and the toe is shown in blue. The CoP model used in this paper began at 25% of the x-distance from MTP to the toe, and moved toward the toe at a rate of *e* ^*x*^, indicated by the solid black line. (PDF 119 kb)

Additional file 3 **Table S1.** Effect of CoP model on muscle stress by joint. Stresses for each model are shown as a proportion of the model used (25% initial location, exponential movement). (PDF 30 kb)

Additional file 4 **Figure S3.** Joint Moment Sensitivity Analysis. The effect of different models of CoP movement on net joint moments with respect to time for all trials. The shaded area represents one standard deviation above and below the mean pattern of joint moment, depicted by bold lines for each model, as noted in the figure panel legends. The CoP model used in this paper began at 25% of the distance from MTP to the toe, and moved toward the toe at a rate of *e* ^*x*^, indicated by the solid line in each plot. (PDF 29 kb)

## Abbreviations

BW: Body weight of jerboa
CoP: Center of pressure
CoM: Center of mass
CSA: Cross-sectional area of tendon (Eq. 3)
GRF: Ground reaction force
MTP: Metatarsal-Phalangeal joint
PCSA: Physiological cross-sectional area of muscle (Eq. 1)
*θ*: Joint angle (Eq. 2)
*ϕ*: Muscle pennation angle
*ρ*_*x*_: Density of *x*
*M*_*x*_: Ground reaction force moment of joint *x*
*F*_*x*_: Force produced by muscle *x*
*r*_*x*_: Moment arm of muscle *x*
*W*: Tendon Spring Energy
*Δ**L*: Change of tendon length
