# Supplementary figures and images for: Vertical leaping mechanics of the Lesser Egyptian Jerboa reveal specialization for maneuverability rather than elastic energy storage
Source: Front Zool. 2017 Jul 3;14:32. doi: 10.1186/s12983-017-0215-z (PMC5496339; doi:10.1186/s12983-017-0215-z)

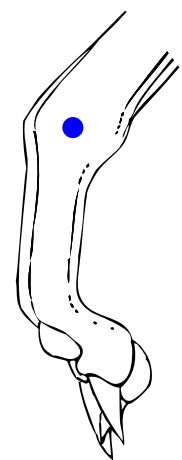

X-distance from Toe (mm)

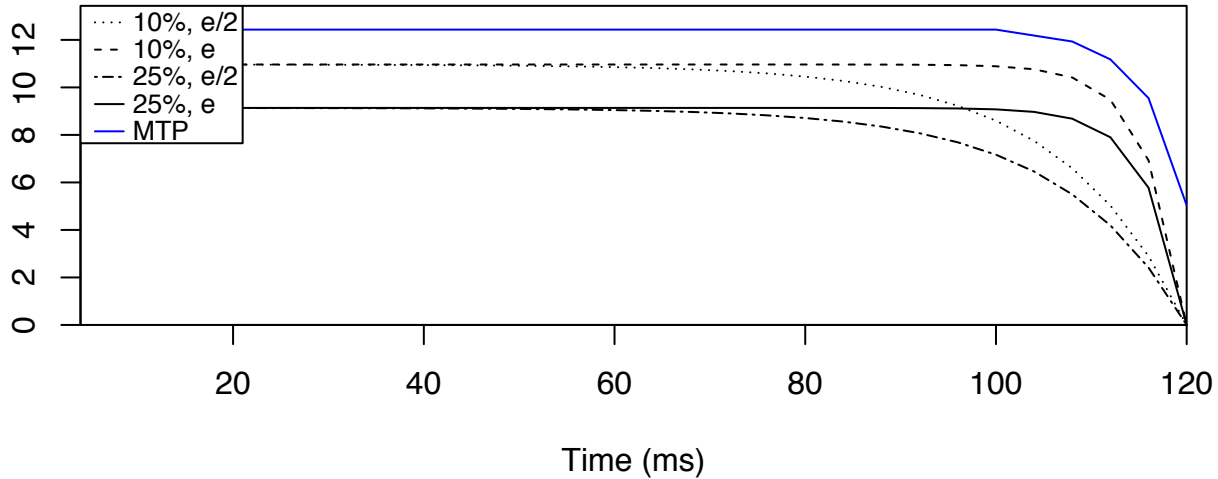

Supplement: Supplementary file 2 — Figure S2. Center of Pressure Sensitivity Analysis. The x-distance between the CoP and the toe through time, in a representative trial. The x-distance between the MTP and the toe is shown in blue. The CoP model used in this paper began at 25% of the x-distance from MTP to the toe, and moved toward the toe at a rate of e x, indicated by the solid black line. (PDF 119 kb) [file 12983_2017_215_MOESM2_ESM.pdf]

**MTP**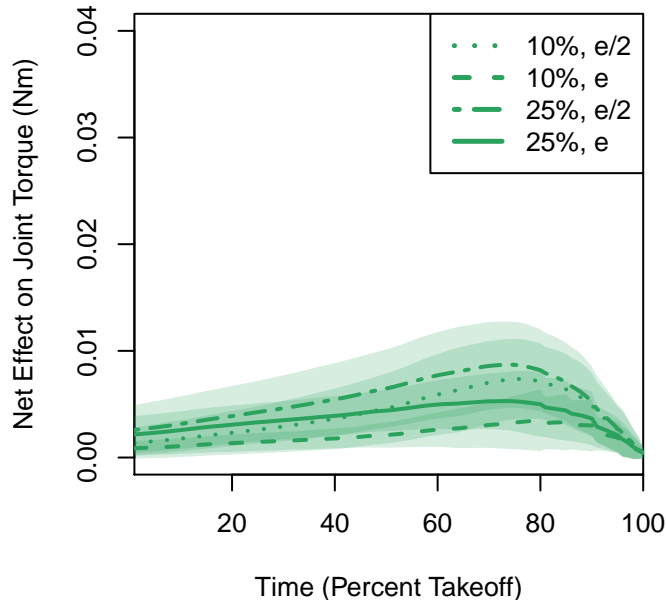**Ankle**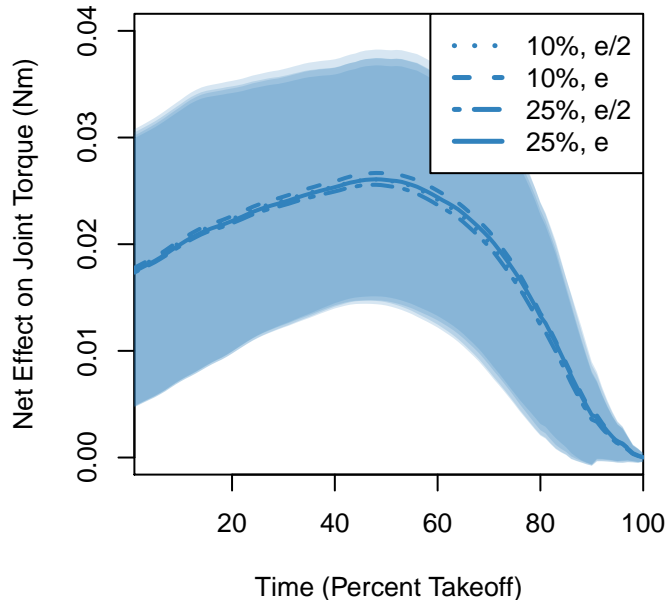**Knee**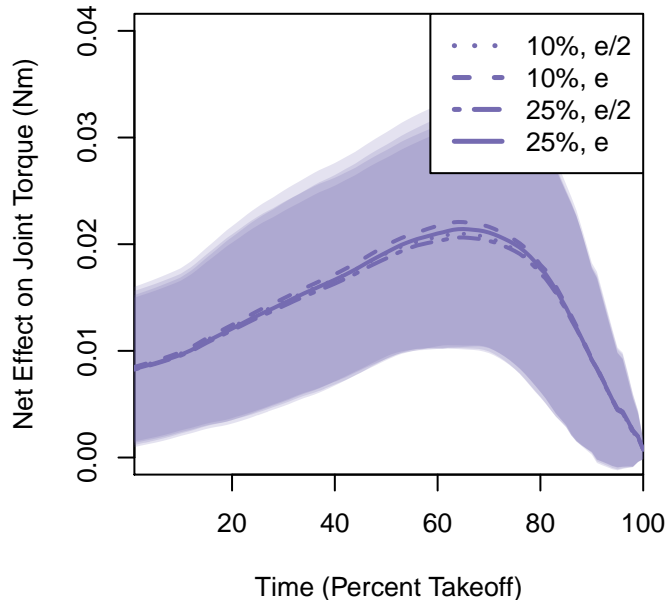**Hip**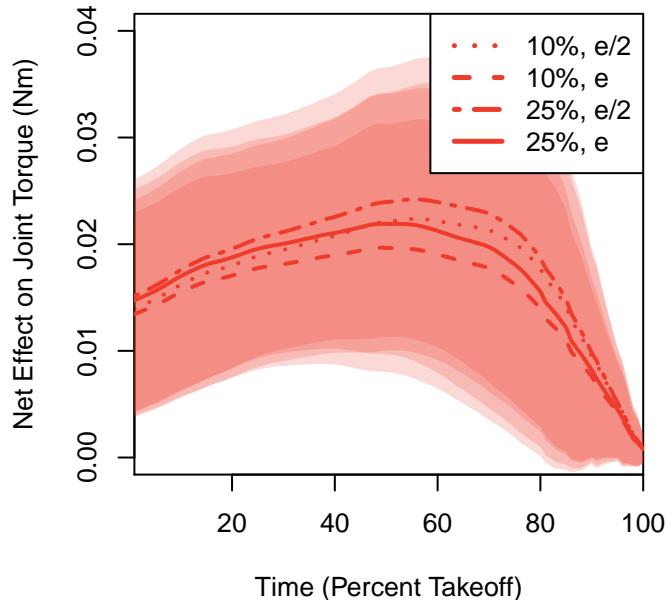

Supplement: Supplementary file 4 — Figure S3. Joint Moment Sensitivity Analysis. The effect of different models of CoP movement on net joint moments with respect to time for all trials. The shaded area represents one standard deviation above and below the mean pattern of joint moment, depicted by bold lines for each model, as noted in the figure panel legends. The CoP model used in this paper began at 25% of the distance from MTP to the toe, and moved toward the toe at a rate of e x, indicated by the solid line in each plot. (PDF 29 kb) [file 12983_2017_215_MOESM4_ESM.pdf]
