# Supplementary material for: Vertical leaping mechanics of the Lesser Egyptian Jerboa reveal specialization for maneuverability rather than elastic energy storage
Source: Front Zool. 2017 Jul 3;14:32. doi: 10.1186/s12983-017-0215-z (PMC5496339; doi:10.1186/s12983-017-0215-z)
Supplement: Supplementary file 3 — Table S1. Effect of CoP model on muscle stress by joint. Stresses for each model are shown as a proportion of the model used (25% initial location, exponential movement). (PDF 30 kb) [file 12983_2017_215_MOESM3_ESM.pdf]

Table S1 Effect of CoP model on muscle stress by joint. Stresses for each model are shown as a proportion of the model used (25% initial location, exponential movement).

Joint	10%, $e/2$	10%, e	25%, $e/2$
MTP	2.01	1.31	2.35
Ankle	0.98	1.00	0.96
Knee	0.96	0.98	0.94
Hip	1.10	1.01	1.18
